# Rapamycin treatment induces tubular proteinuria: role of megalin-mediated protein reabsorption

**DOI:** 10.3389/fphar.2023.1194816

**Published:** 2023-07-07

**Authors:** Rodrigo A. S. Peres, Diogo B. Peruchetti, Rodrigo P. Silva-Aguiar, Douglas E. Teixeira, Carlos P. Gomes, Christina M. Takiya, Ana Acacia S. Pinheiro, Celso Caruso-Neves

**Affiliations:** ^1^ Carlos Chagas Filho Institute of Biophysics, Federal University of Rio de Janeiro, Rio de Janeiro, Brazil; ^2^ Department of Physiology and Biophysics, Federal University of Minas Gerais, Belo Horizonte, Brazil; ^3^ Clementino Fraga Filho University Hospital, Federal University of Rio de Janeiro, Rio de Janeiro, Brazil; ^4^ School of Medicine and Surgery, Federal University of the State of Rio de Janeiro, Rio de Janeiro, Brazil; ^5^ Rio de Janeiro Innovation Network in Nanosystems for Health-NanoSAÚDE/FAPERJ, Rio de Janeiro, Brazil; ^6^ National Institute of Science and Technology for Regenerative Medicine, Rio de Janeiro, Brazil

**Keywords:** rapamycin, proximal tubule, megalin, protein reabsorption, proteinuria

## Abstract

**Introduction:** Rapamycin is an immunosuppressor that acts by inhibiting the serine/threonine kinase mechanistic target of rapamycin complex 1. Therapeutic use of rapamycin is limited by its adverse effects. Proteinuria is an important marker of kidney damage and a risk factor for kidney diseases progression and has been reported in patients and animal models treated with rapamycin. However, the mechanism underlying proteinuria induced by rapamycin is still an open matter. In this work, we investigated the effects of rapamycin on parameters of renal function and structure and on protein handling by proximal tubule epithelial cells (PTECs).

**Methods:** Healthy BALB/c mice were treated with 1.5 mg/kg rapamycin by oral gavage for 1, 3, or 7 days. At the end of each treatment, the animals were kept in metabolic cages and renal function and structural parameters were analyzed. LLC-PK1 cell line was used as a model of PTECs to test specific effect of rapamycin.

**Results:** Rapamycin treatment did not change parameters of glomerular structure and function. Conversely, there was a transient increase in 24-h proteinuria, urinary protein to creatinine ratio (UPCr), and albuminuria in the groups treated with rapamycin. In accordance with these findings, rapamycin treatment decreased albumin-fluorescein isothiocyanate uptake in the renal cortex. This effect was associated with reduced brush border expression and impaired subcellular distribution of megalin in PTECs. The effect of rapamycin seems to be specific for albumin endocytosis machinery because it did not modify renal sodium handling or (Na^+^+K^+^)ATPase activity in BALB/c mice and in the LLC-PK1 cell line. A positive Pearson correlation was found between megalin expression and albumin uptake while an inverse correlation was shown between albumin uptake and UPCr or 24-h proteinuria. Despite its effect on albumin handling in PTECs, rapamycin treatment did not induce tubular injury measured by interstitial space and collagen deposition.

**Conclusion:** These findings suggest that proteinuria induced by rapamycin could have a tubular rather than a glomerular origin. This effect involves a specific change in protein endocytosis machinery. Our results open new perspectives on understanding the undesired effect of proteinuria generated by rapamycin.

## 1 Introduction

Rapamycin, a macrolide compound, was purified in 1975 from bacteria isolated in the soil of Rapa Nui Island and described to have an antifungal effect (Vézina et al., 1975). Later, it was shown that rapamycin has anti-proliferative and immunosuppressor effects (Martel et al., 1977). Rapamycin inhibits serine/threonine kinase mechanistic target of rapamycin (mTOR), which belongs to the phosphatidylinositol-3 kinase (PI3K)-related protein kinase family (Sabatini et al., 1994; Sabers et al., 1995). Rapamycin has been reported to promote specific inhibition of mechanistic target of rapamycin (mTOR) complex 1 (mTORC1) through its binding to prolyl-isomerase FK506 binding protein 12 (FKBP12), which interacts with the FKBP-rapamycin-binding domain in mTOR, obstructing substrate access to the kinase active site (Liu and Sabatini, 2020). Nowadays, it is known that chronic use of rapamycin could also inhibit mTORC2 (Sarbassov et al., 2006). Due to its immunosuppressor effect, the use of rapamycin was first approved for the treatment of patients after kidney transplant as an alternative to the nephrotoxic effect of other immunosuppressors (Johnson et al., 2001; Chueh and Kahan, 2005; Weir et al., 2011).

The mTOR pathway is involved in the genesis of different pathologies (Fantus et al., 2016; Gui and Dai, 2020; Liu and Sabatini, 2020), and the use of rapamycin was proposed for the treatment of heart, kidney, and autoimmune diseases (McMullen et al., 2004; Chen et al., 2012; Cui et al., 2015; Shao et al., 2017; Gao et al., 2020; Peng et al., 2020). Although it has therapeutic effects, the use of rapamycin is associated with adverse effects (Tedesco-Silva et al., 2019). Clinical studies investigating the effects of rapamycin and its analogs in patients after heart and kidney transplant have reported the development of proteinuria (van den Akker et al., 2006; Aliabadi et al., 2008; Wiseman et al., 2013; Tedesco-Silva et al., 2019; Asleh et al., 2021). Development of proteinuria has also been shown in healthy murine models treated with rapamycin (Stylianou et al., 2012; Gleixner et al., 2014). Proteinuria is an important marker of kidney damage and a risk factor for renal disease progression (Cravedi and Remuzzi, 2013), which limits the therapeutic use of rapamycin. However, the mechanism underlying the effect of rapamycin on proteinuria is still an open matter (Rangan, 2006; Straathof-Galema et al., 2006; van den Akker et al., 2006).

Proteinuria involves changes in glomerular permeability and/or proximal tubule reabsorption (Haraldsson et al., 2008; Dickson et al., 2014; Eshbach and Weisz, 2017). Pharmacologic and genetic approaches to inhibit mTORC1 activation have shown the involvement of mTOR in the regulation of both glomerular membrane permeability and protein endocytosis in proximal tubule epithelial cells (PTECs) (Vollenbroker et al., 2009; Gödel et al., 2011; Grahammer et al., 2016). Under physiologic conditions, proteins filtered through the glomerulus are completely reabsorbed by PTECs through clathrin-dependent receptor-mediated endocytosis (Christensen et al., 2012; Eshbach and Weisz, 2017). This endocytic pathway relies on the expression and localization of megalin, a scavenger receptor that forms a multiligand receptor complex with cubilin and amnionless at the brush border membrane (Christensen et al., 2012; Eshbach and Weisz, 2017). Megalin is crucial for the internalization of this complex with protein targeted on lysosome degradation and megalin recycled to the surface membrane (Marzolo and Farfán, 2011; Perez Bay et al., 2016). The impairment of megalin-mediated protein endocytosis in PTECs results in tubular proteinuria and is associated with the development of tubule interstitial injury (Nielsen et al., 2016; Peruchetti et al., 2020; Peruchetti et al., 2021; Peres et al., 2023). In addition, it has been shown that the expression of megalin in PTECs is modulated by the mTOR pathway (Gleixner et al., 2014; Peruchetti et al., 2014).

Thus, it is plausible to postulate that proteinuria induced by rapamycin treatment could be associated, at least in part, with changes in megalin-mediated protein reabsorption in PTECs. In the present work, we investigated the effects of rapamycin on parameters of renal function and structure as well as on protein handling by PTECs. To address this question, BALB/c mice and LLC-PK1 cells, a model of PTECs, were used. Our results show that rapamycin treatment reduced megalin-mediated protein endocytosis in PTECs, leading to tubular proteinuria without any change in glomerular function. This effect involves a decrease in megalin expression and cellular distribution.

## 2 Materials and methods

### 2.1 Reagents

Acrylamide, ATP (sodium salt), bovine serum albumin (BSA) fraction V (#A9647), BSA conjugated to fluorescein isothiocyanate (BSA-FITC), bromophenol blue, 2-mercaptoethanol, phenylmethylsulfonyl fluoride (PMSF), D-glucose, protease inhibitor cocktail (ref. I3786), MOPS, HEPES, EDTA, sucrose, Triton X-100, Tween 20, sodium fluoride, sodium pyrophosphate, sodium orthovanadate, sodium β-glycerophosphate, trichloroacetic acid, tetramethylethylenediamine (TEMED), periodic acid-Schiff (PAS) reagent, ouabain (O3125), Sirius red, Harry’s hematoxylin, Folin and Ciocalteu’s phenol reagent were purchased from Sigma-Aldrich (St. Louis, MO, United States). Polyvinylidene fluoride (PVDF) membranes and methanol were purchased from Merck Millipore (Barueri, SP, Brazil). ECL Prime, sodium dodecyl sulfate, and Tris were purchased from GE Healthcare (Pittsburgh, PA, United States). Dulbecco’s modified Eagle’s medium (DMEM), phosphate-buffered saline (PBS), fetal bovine serum (FBS), antibiotic-antimycotic (100×), 4′,6-diamidino-2-phenylindole (DAPI), and UltraPure N,N′-methylenebisacrylamide (bisacrylamide) were purchased from Thermo Fisher Scientific (Waltham, MA, United States). Sodium deoxycholate, 1-amino-2-hydroxy-4-nathtalene sulfonic acid, sodium bisulfite, sodium sulfite, sodium chloride, potassium chloride, magnesium chloride, monosodium phosphate monohydrated, and disodium phosphate were purchased from VETEC (Duque de Caxias, RJ, Brazil). The LLC-PK1 cell line was purchased from the American Type Culture Collection (ATCC) (Manassas, VA, United States). ^32^Pi was obtained from the Brazilian Institute of Energetic and Nuclear Research (São Paulo, SP, Brazil). Rapamycin was purchased from Pfizer (Itapevi, SP, Brazil). Polyclonal Lrp2/megalin (ab76969) and monoclonal albumin (ab207327) antibodies were purchased from Abcam (Cambridge, MA, United States). Anti-rabbit IgG HRP (#7074) was purchased from Cell Signal Technologies (Danvers, MA, United States). Fluorescent anti-rabbit IgG Alexa Fluor Plus 594 (A32754) and anti-rabbit IgG Alexa Fluor Plus 488 (A32731) were purchased from Thermo Fisher Scientific.

### 2.2 Animals

Male BALB/c mice (8–10 weeks old), weighing 20–24 g, were used in all experiments. All mice were obtained from The Animal Care Facility of the Health Science Center of the Universidade Federal do Rio de Janeiro (UFRJ), Rio de Janeiro, Brazil. The mice were accommodated in an air-conditioned environment (22°C–24°C) with a regular 12-h light/dark cycle and water and standard chow *ad libitum*. The handling and experimental procedures were conducted in accordance with the National Institutes of Health (NIH) Guide for the Care and Use of Laboratory Animals and were approved by the Institutional Ethics Committee of the UFRJ (CEUA 045/17).

### 2.3 Rapamycin treatment

Animals were treated with daily doses of rapamycin (1.5 mg/kg) by oral gavage (200 µL) for different times. The animals were randomly divided into four experimental groups: (1) CTL (control), animals received a daily dose of water (used as vehicle); (2) D1, animals received a single dose of rapamycin; (3) D3, animals received daily doses of rapamycin for 3 consecutive days; (4) D7, animals received daily doses of rapamycin for 7 consecutive days. At the end of each experimental period, the mice were kept in metabolic cages for 24-h urine collection. The animals were then euthanized using a mixture of ketamine (240 mg/kg) and xylazine (15 mg/kg), followed by cardiac puncture for blood collection to obtain plasma. Urine and plasma samples were used for analysis of renal function. Urine samples were also used to determine urinary proteins and albuminuria using SDS-PAGE followed by immunoblotting. The kidneys were perfused with heparinized saline and extracted for further analysis: (1) histology; (2) immunofluorescence; (3) *in vivo* albumin endocytosis; (4) renal (Na^+^+K^+^)ATPase activity assay.

### 2.4 Analysis of renal function

Analysis of renal function was performed according to previously published studies (Silva-Aguiar et al., 2018; Teixeira et al., 2020; Peruchetti et al., 2021; Farias et al., 2023; Peres et al., 2023). Briefly, 24-h urine samples were quantified to determine urinary volume (mL) and urinary flow (mL/min). Urine samples were then centrifuged 5 times (10,000 × *g* for 10 min) to remove urine sediments. Plasma was obtained by centrifuging whole blood (2,500 × *g* for 5 min). All parameters analyzed in urine and plasma were measured using commercial kits following the manufacturers’ instructions. The levels of creatinine, blood urea nitrogen (BUN), and urinary proteinuria in both urine and plasma were measured, respectively, using the creatinine kit (ref. 35-100), urea CE kit (ref. 27-500), and sensiprot kit (ref. 36) purchased from Labtest (Lagoa Santa, MG, Brazil). The levels of urinary and plasma sodium were measured using the enzymatic sodium kit (ref. BT1201100) purchased from BioTecnica (Varginha, MG, Brazil). These results were then utilized to calculate parameters such as creatinine clearance (CCr), sodium clearance, renal fractional excretion of sodium (FE_Na_^+^), and urinary protein to creatinine ratio (UPCr).

### 2.5 Histologic analysis

Histologic analysis was performed according to previously published studies (Silva-Aguiar et al., 2018; Teixeira et al., 2020; Peruchetti et al., 2021; Farias et al., 2023; Peres et al., 2023). Briefly, perfused kidneys were fixed in a 10% formalin buffer solution for 24 h and embedded in paraffin. Slices of paraffin-embedded kidneys (5–8 µm thick) were stained with periodic acid-Schiff (PAS) and Picrosirius red. Images of the renal cortex were acquired using a Nikon 80i eclipse microscope (Nikon, Japan) and analyzed in a blinded manner using Image-Pro Plus Software (Media Cybernetics, Rockville, MD, United States). Glomerular area (pixels/area) was defined as the area delimited by the outer side of Bowman’s capsule. Glomerular cellularity (cells/tuft area) was determined by counting stained nuclei in the glomerular tuft area. The cortical tubule interstitial area was analyzed by directly measuring the area between cortical tubules divided by the total area of cortical tubules (% of total area). Collagen deposition (% of total area) was analyzed by measuring the intensity of red fibers in selected hot spot areas divided by the total area of tubules in the field.

### 2.6 Cell culture

LLC-PK1 cells, a well-established porcine PTEC line (Hull et al., 1976; Takakura et al., 1995; Nielsen et al., 1998) were cultured in low-glucose DMEM supplemented with 10% FBS and 1% penicillin/streptomycin at 37°C in humidified air containing 5% CO_2_, as described previously (Teixeira et al., 2019; Peruchetti et al., 2020; Peruchetti et al., 2021; Silva-Aguiar et al., 2022a,b; Peres et al., 2023). Cells were seeded in 24-well plates and grown for 2 days until 85%–90% confluence was reached. Cells were maintained overnight in serum-depleted medium. When indicated, the cells were incubated with rapamycin at 10^–7^, 10^–8^, and 10^–9^ M to determine (Na^+^+K^+^)ATPase activity in the cell lysate and LDH activity in the cell supernatant. Lactate dehydrogenase (LDH) was measured using a LDH liquiform kit (ref. 86-2/30) purchased from Labtest (Lagoa Santa, MG, Brazil).

The cells were seeded on glass cover slips for analysis of immunofluorescence. The assessment of albumin endocytosis and megalin expression was performed in cells incubated with rapamycin at 10^–7^ M.

### 2.7 Immunofluorescence and confocal microscopy

Immunofluorescence analyses were performed on kidney tissue and LLC-PK1 cells in accordance with previously published studies (Silva-Aguiar et al., 2018; Teixeira et al., 2020; Peruchetti et al., 2021; Farias et al., 2023; Peres et al., 2023). Briefly, immunofluorescence analysis of megalin was performed using 5-µm-thick kidney slices prepared as described earlier and LLC-PK1 cells grown on cover slips, fixed with 4% paraformaldehyde and permeabilized with PBS (136 mM NaCl, 2.7 mM KCl, 8 mM Na_2_HPO_4_, 1.76 mM KH_2_PO_4_) containing 0.1% Triton X-100. Samples were blocked with 5% BSA at room temperature for 1 h. Polyclonal anti-rabbit megalin antibody (1:100) was incubated overnight at 4°C. Secondary antibody incubation was performed at room temperature for 1 h with fluorescent anti-rabbit IgG Alexa Fluor 594 (1:200) for kidney samples and anti-rabbit IgG Alexa Fluor 488 (1:200) for cell samples. Cell nuclei were stained with DAPI (1 μg/mL) for 5 min at room temperature. The tissue slices and cover slips were mounted with anti-fade mounting medium. Images were visualized and acquired by confocal microscopy (Leica TCS SP8, Leica, Wetzlar, Germany) and analyzed using FIJI software version 2.1.0 (NIH, Bethesda, MD, United States). To highlight megalin expression in proximal tubules (PTs), 3D projection was performed with the Interactive 3D Surface Plot plugin v2.4.1, and the signal intensity was highlighted by applying a colorized Thermal Look-Up Table on selected PT regions. To analyze the apical-to-basolateral distribution of megalin, a plot profile of the signal intensities was generated by drawing a straight line from the apical to the basolateral side of individual cells on PT segments (Schuh et al., 2018; Peres et al., 2023). Based on each plot profile curve generated, the megalin expression in the brush border was quantified by calculating the area under the curve (AUC) of the apical area. The ratio between brush border and basolateral megalin expression was calculated by dividing the apical AUC by the basolateral AUC (brush border/basolateral). Megalin expression in LLC-PK1 cells was quantified by measuring the signal intensity corrected by the cell area.

### 2.8 (Na^+^+K^+^)ATPase activity

(Na^+^+K^+^)ATPase activity was measured in renal cortex homogenates and cell lysates as published previously (Queiroz-Madeira et al., 2010; Peruchetti et al., 2011; Arnaud-Batista et al., 2016; Silva et al., 2018; Teixeira et al., 2020). To obtain the tissue homogenate, the renal cortex of extracted kidneys was isolated and homogenized in ice-cold Ringer solution (20 mM HEPES-Tris [pH 7.4], 5 mM D-glucose, 2.7 mM KCl, 140 mM NaCl, 1 mM MgCl_2_, 1.8 mM CaCl_2_) containing 1 mM PMSF and 1× protease inhibitor cocktail. Next, the samples were clarified by centrifugation (10,000 × *g* for 10 min at 4°C) and the supernatant was stored at −80°C until required. To obtain LLC-PK1 cell lysates, the cells were washed twice with ice-cold PBS solution and lysed with lysis buffer (1 mM EGTA, 0.1% sodium deoxycholate, 20 mM HEPES-Tris [pH 7.0], 250 mM sucrose). The lysates were clarified twice by centrifugation (10,000 × *g* for 10 min at 4°C) and the supernatant was collected to determine the enzyme activity. ATPase activity was measured in standard reaction medium containing 4 mM MgCl_2_, 5 mM ATP (specific activity 0.27 μCi/nmol [γ^32^P]ATP), 20 mM HEPES-Tris (pH 7.0), 120 mM NaCl, 30 mM KCl. [γ^32^P]ATP was used as a tracer. To determine the specific (Na^+^+K^+^)ATPase activity, the standard reaction medium was supplemented with 1 mM ouabain, a specific inhibitor of (Na^+^+K^+^)ATPase. The reaction was started by adding protein samples to a final concentration of 0.5 mg/mL kidney homogenate and/or 0.3–0.5 mg/mL cell lysate to the standard reaction medium and incubating at 37°C for 20 min. The reaction was stopped with ice-cold charcoal activated by 0.1 N HCl and centrifuged for 5 min at 1,255 × *g*. The supernatant was collected and the ^32^Pi released was measured using a liquid scintillation counter (Packard Tri-Carb 2100 TR). The specific (Na^+^+K^+^)ATPase activity was calculated by the difference between the ^32^Pi released in the reaction with and without 1 mM ouabain. Results are expressed as nanomoles Pi per milligram of protein per minute.

### 2.9 Urine SDS-PAGE and immunoblotting analysis

Urine SDS-PAGE and immunoblotting were performed as published previously (Silva-Aguiar et al., 2018; Teixeira et al., 2020; Peruchetti et al., 2021; Farias et al., 2023; Peres et al., 2023). Briefly, 24-h urine samples were centrifuged 5 times (10,000 × *g* for 10 min) to remove urine sediments. The urinary creatinine concentration was determined as described above for sample normalization. Then, urine samples were resolved on 12% SDS-PAGE and transferred to PVDF membranes. The membranes were blocked with 5% milk and incubated with monoclonal anti-albumin antibody overnight at 4°C; secondary antibodies were incubated for 1 h at room temperature. ECL Prime was used for detection. Urine SDS-PAGE gels were stained with 0.125% Coomassie brilliant blue R-250 overnight at 4°C to analyze the urinary protein profile. All the images were obtained using the Image Quant LAS4000 Image processing system (GE Healthcare Life Sciences, Pittsburgh, PA, United States). The images were prepared with FIJI software version 2.1.0 (NIH, Bethesda, MD, United States).

### 2.10 Albumin-FITC uptake *in vivo*

Albumin-FITC uptake *in vivo* was measured in the renal cortex as published previously (Silva-Aguiar et al., 2018; Teixeira et al., 2020; Peruchetti et al., 2021; Farias et al., 2023; Peres et al., 2023). Briefly, a solution of BSA-FITC (5 μg/g body weight) was injected into mice through the tail vein. After 15 min, the animals were euthanized, and the kidneys were perfused with heparinized saline and extracted. The renal cortex was isolated and homogenized as described earlier. Kidney homogenates were centrifuged (10,000 × *g* for 10 min) for clarification. The cortex-associated fluorescence was measured in the supernatant using a SpectraMax M2 microplate reader (excitation, 480 nm; emission, 520 nm) (Molecular Devices, San Jose, CA, United States). The total protein concentration of each sample was determined by the Folin phenol method (Lowry et al., 1951). The specific cortical albumin-FITC uptake was corrected by the total protein concentration of each sample.

### 2.11 Albumin endocytosis *in vitro*

*In vitro* albumin endocytosis was performed according to previous studies (Peruchetti et al., 2018; Peruchetti et al., 2021; Silva-Aguiar et al., 2022a,b; Peres et al., 2023). After rapamycin treatment, the cells were washed three times and incubated with Ringer solution containing 100 μg/mL BSA-FITC at 37°C for 30 min. After incubation, the cells were kept on ice, and unbound BSA-FITC was removed by washing the cells ten times with ice-cold Ringer solution. Cells were lysed with a buffered detergent solution containing 20 mM MOPS (pH 7.4) and 0.1% Triton X-100. The homogenate was collected to measure the cell-associated fluorescence using a SpectraMax M2 microplate reader (Molecular Devices). The specific fluorescence of endocytosed BSA-FITC was obtained by subtracting non-specific fluorescence of BSA-FITC uptake determined on cells co-incubated with unlabeled BSA (100 mg/mL). The specific cell-associated fluorescence was further normalized to the total protein concentration of each sample.

### 2.12 Statistical analysis

All results are presented as medians (interquartile range). The Shapiro-Wilk test was used to evaluate normal data distribution. To compare differences between groups, one-way analysis of variance (ANOVA) was used followed by Tukey’s post-test. When indicated, the *t*-test was used. Pearson’s coefficient was used to identify correlations between sets of data. *p* < 0.05 was considered statistically significant. The statistical analysis was performed using GraphPad Prism (version 8; GraphPad Software, San Diego, CA, United States).

## 3 Results

### 3.1 Rapamycin treatment induced no changes in glomerular function and structure

**FIGURE 1:**
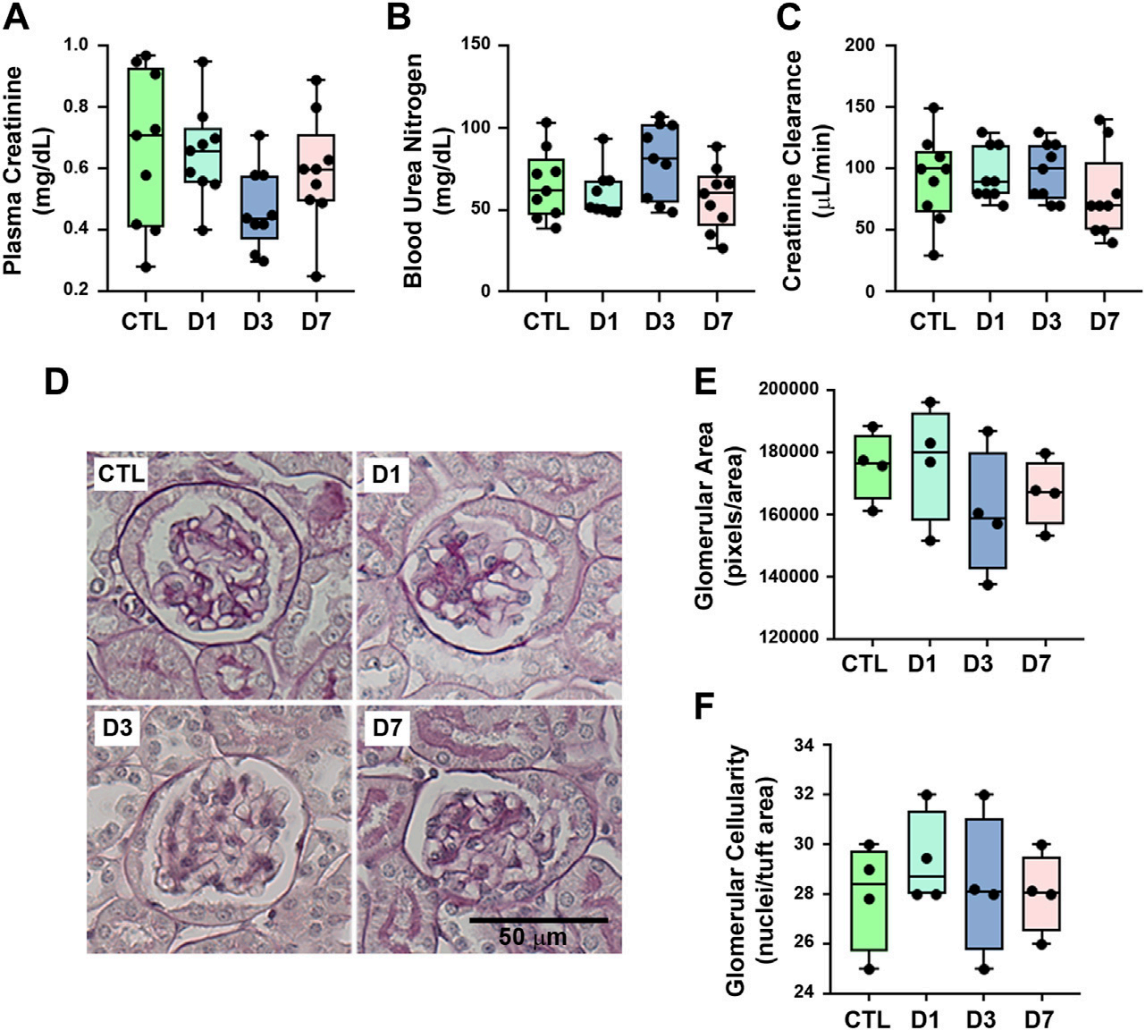
Rapamycin treatment does not change glomerular function and structure. Functional and structural analyses were performed on each experimental group as described in Section 2. **(A)** Plasma creatinine (*n* = 9). **(B)** Blood urea nitrogen (BUN) (*n* = 9). **(C)** Creatinine clearance (*n* = 9). **(D)** Representative micrographs of cortical glomerulus in kidney sections stained with periodic acid-Schiff, Scale bar, 50 µm. **(E)** Quantitative analysis of glomerular area (pixels/area) (*n* = 4). **(F)** Glomerular cellularity (cells/tuft area) (*n* = 4). In panel **(E)** and **(F)**, each data point represents the mean value obtained from measurements of 15 different glomeruli. Data are presented as medians (interquartile range).

To evaluate the possible effects of rapamycin on the renal function parameters, BALB/c mice were treated with rapamycin for different times generating four groups: a) control (CTL), animals did not receive rapamycin; b) D1, D3, and D7, animals received a daily dose of rapamycin for 1, 3 or 7 days, respectively. Body weight, kidney weight/body weight ratio, food intake, water intake, urinary flow, and urinary creatinine did not change between the groups (Table 1). Markers of glomerular function such as plasma creatinine, BUN, and creatinine clearance (CCr) showed no difference between the groups (Figures 1A–C). In addition to the functional analysis, the assessment of PAS-stained kidney slices showed no differences in the glomerular area and cellularity between rapamycin-treated animals and controls (Figures 1D–F). These results suggest that glomerular function and structure were not changed by rapamycin treatment.

**TABLE 1:** Functional parameters (mean ± standard deviation).

Parameters	Control (*n* = 9)	Day 1 (*n* = 9)	Day 3 (*n* = 9)	Day 7 (*n* = 9)
Body weight (g)	23.14 ± 2.26	21.89 ± 1.54	20.70 ± 2.60	22.31 ± 1.77
Kidney weight/body weight (mg/g)	8.67 ± 0.86	9.44 ± 0.44	8.87 ± 0.92	9.53 ± 0.0.83
Food intake (g/24 h)	4.13 ± 0.63	3.32 ± 0.73	4.19 ± 0.90	4.27 ± 0.60
Water intake (mL/24 h)	6.05 ± 2.06	6,04 ± 0.96	4.80 ± 2.11	5.44 ± 1.33
Urinary flow (μL/min)	0.61 ± 0.24	0.62 ± 0.15	0.62 ± 0.14	0.55 ± 0.21
Urinary creatinine (mg/dL)	91.61 ± 13.70	90.39 ± 9.11	75.71 ± 11.01	84.87 ± 18.37

### 3.2 Rapamycin treatment induces tubular proteinuria and albuminuria

**FIGURE 2:**
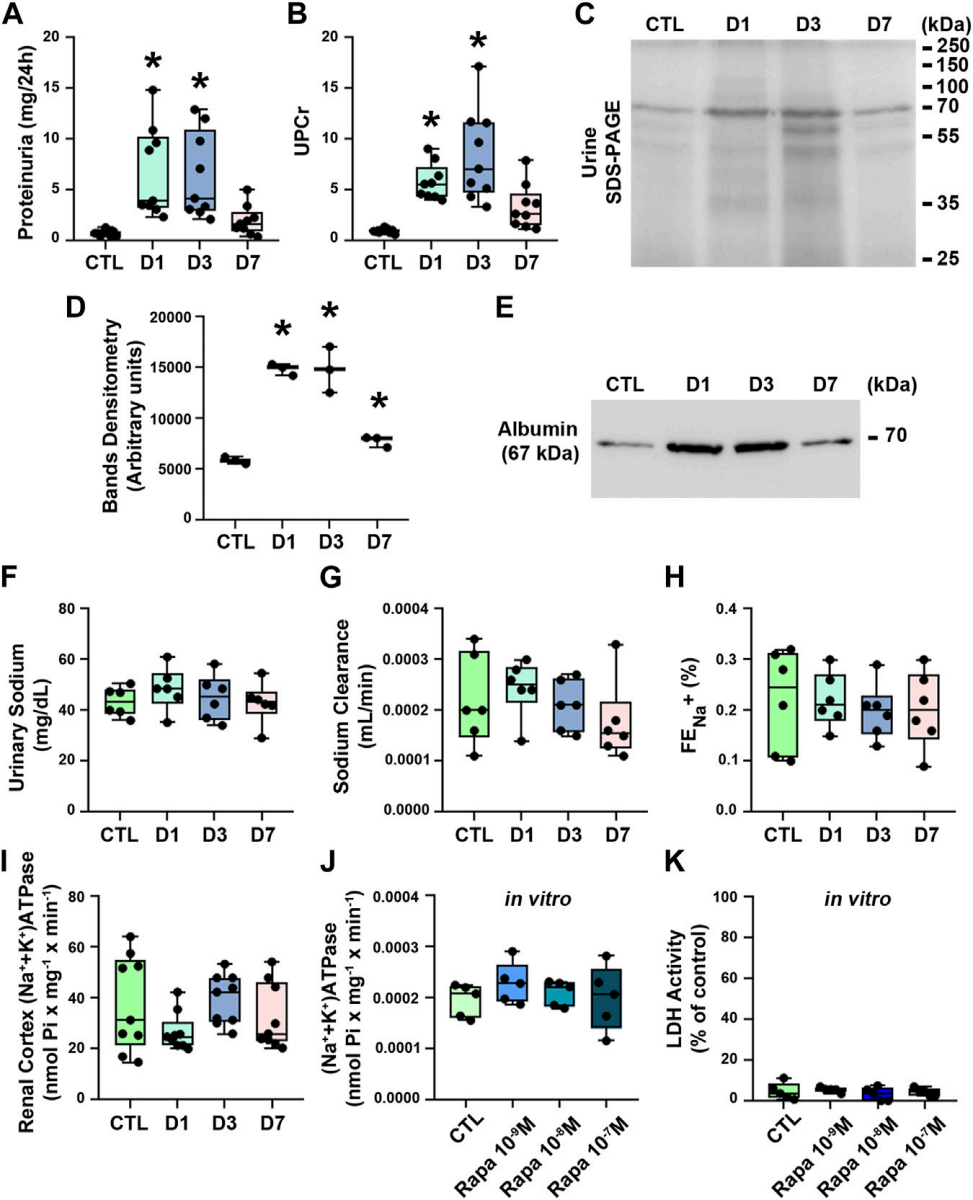
Rapamycin treatment induces proteinuria and albuminuria without changes in renal sodium handling. **(A)** 24-h proteinuria (mg/24 h) (*n* = 9). **(B)** Urinary protein creatinine ratio (UPCr) (*n* = 9). **(C)** Assessment of urinary proteins by SDS-PAGE stained with Coomassie blue. Representative image of three independent experiments. **(D)** Densitometry analysis of urinary protein from SDS-PAGE. **(E)** Representative immunoblotting image of urinary albumin (67 kDa). **(F)** Urinary sodium (*n* = 6). **(G)** Sodium clearance (*n* = 6). **(H)** Renal fractional excretion of sodium (FE_Na_^+^) (*n* = 6). **(I)** (Na^+^+K^+^)ATPase activity in renal cortex (*n* = 9). **(J)** (Na^+^+K^+^)ATPase activity in LLC-PK1 cells (*n* = 5). **(K)** Lactate dehydrogenase (LDH) activity in the supernatant of LLC-PK1 cells (*n* = 5). Data are presented as medians (interquartile range). *p < 0.05 in relation to the control (CTL) group.

To further characterize the effect of rapamycin, the urinary protein excretion as well as proximal tubule function and structure were analyzed. Rapamycin treatment resulted in an increase in 24-h proteinuria and UPCr compared with the control group (Figures 2A,B). The SDS-PAGE analysis of urine showed a profile of middle- and low-molecular weight proteinuria, especially in the D1 and D3 groups compared with the controls (Figures 2C,D). Consistent with these findings, immunoblotting for urinary albumin analysis revealed an increase in albuminuria mainly in the D1 and D3 groups (Figure 2E). There was a tendency to a reduction in proteinuria, UPCr, as well as albuminuria in the D7 group, although they were higher compared with the control group.

Rapamycin treatment did not change renal sodium handling, measured by urinary sodium excretion, sodium clearance and FE_Na_^+^ (Figures 2F–H). In agreement, rapamycin treatment did not change (Na^+^+K^+^)ATPase activity (Figure 2I). Furthermore, we also used LLC-PK1 cells, a model of PTECs, to verify the direct effect of rapamycin on these cells. Overnight treatment of the cells with 10^–7^ M rapamycin did not change (Na^+^+K^+^)ATPase activity and cellular viability measured by LDH activity in the cell supernatant (Figures 2J,K).

**FIGURE 3:**
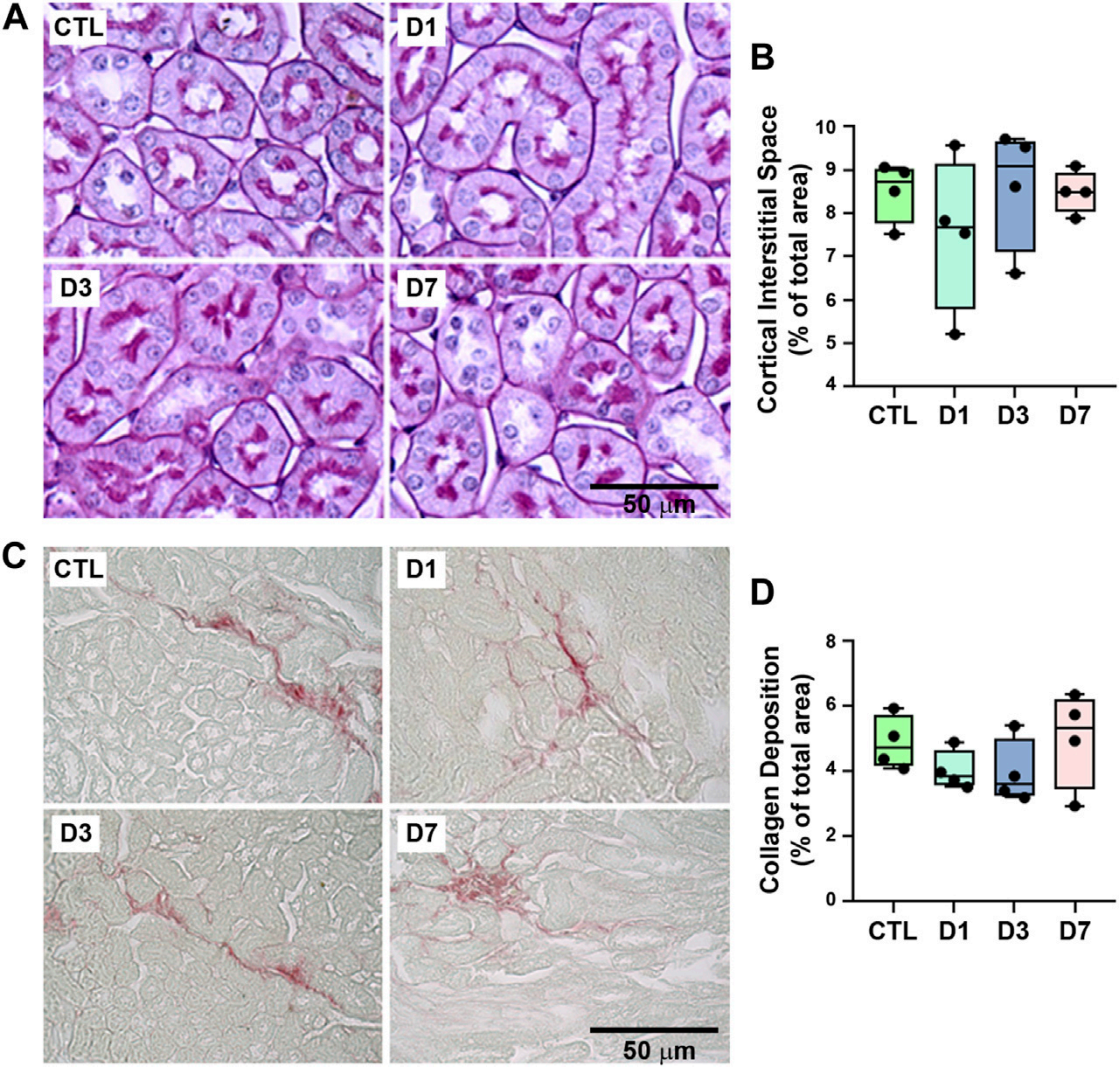
Proximal tubule structure and collagen deposition were not changed by rapamycin treatment. **(A)** Representative micrographs of cortical tubules in kidney sections stained with periodic acid-Schiff. Scale bar, 20 µm. **(B)** Quantitative analysis of tubule interstitial space (% of total area) (*n* = 4). **(C)** Representative micrographs of hotspots of collagen deposition in Picrosirius-stained kidney sections. Scale bar, 50 µm. **(D)** Quantitative analysis of collagen deposition (% of total area) (*n* = 4). In panel B and D, each data point represents the mean value obtained from measurements of 20 different image fields. Data are presented as medians (interquartile range).

The possible effect of rapamycin treatment on tubular injury was assessed using histologic analysis of PAS- and Picrosirius-stained kidney sections, which showed that rapamycin did not induce structural changes in renal cortical tubules, including tubule interstitial space and collagen deposition (Figures 3A–D). These results suggest that rapamycin has a specific effect on tubular protein handling.

### 3.3 Rapamycin treatment reduces albumin-FITC uptake and megalin expression in PTECs

**FIGURE 4:**
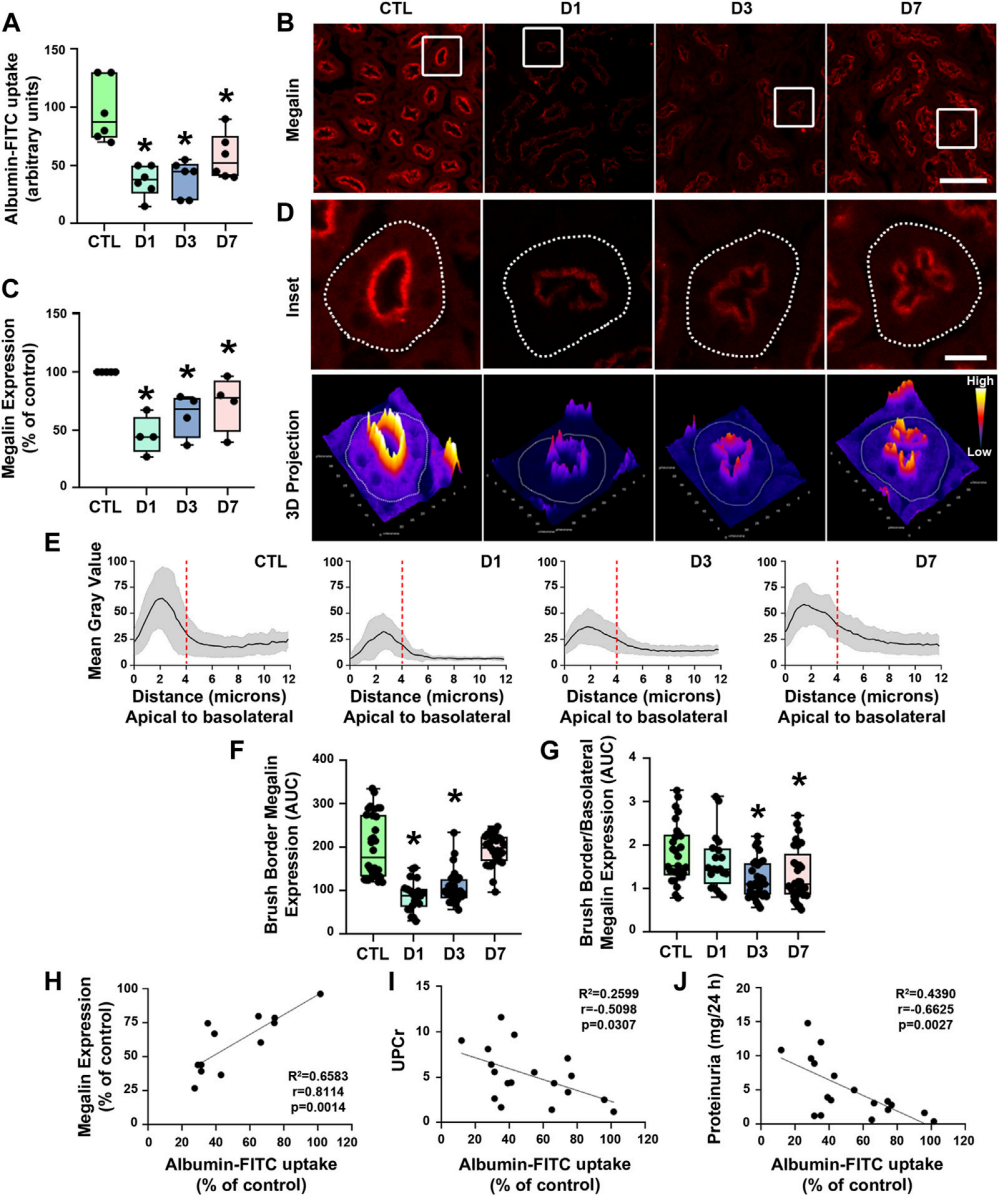
Rapamycin treatment decreases proximal tubule megalin-mediated albumin uptake. **(A)** Albumin-FITC uptake in renal cortex (*n* = 6). **(B)** Representative confocal microscopy images of megalin expression (red) in cortical proximal tubule epithelial cells (PTECs) assessed by immunofluorescence. Scale bar, 50 µm. **(C)** Quantification of total megalin expression in PTECs (*n* = 4). **(D)** Representative confocal microscopy images of apical megalin expression in cortical PTECs (upper panel) and 3D projection of tubular megalin expression (lower panel). Scale bar, 12 µm. **(E)** Plot profile analysis of apical-to-basolateral membrane distribution of megalin in the PTECs as described in Section 2 (*n* = 4, curves represent the sum of plot profile peaks from randomly selected PTECs from different animals). Vertical red dashed lines represent the expected length of the proximal tubule brush border (∼4 µm). **(F)** Apical megalin expression quantified by the area under the curve (AUC) of representative plot profile peaks from each group (*n* = 4, symbols represent the number of different plot profiles analyzed from different cells from each different experimental set). **(G)** Ratio of apical and basolateral megalin expression (*n* = 4, symbols represent the number of different plot profiles analyzed from different cells from each different experimental set). **(H–J)** Correlation between cortical albumin uptake and megalin expression **(H)** or urinary protein to creatinine ratio (UPCr) **(I)** or 24-h proteinuria **(J)**. The data used for analysis are from the D1, D3, and D7 groups. The correlation between variables was calculated using Pearson’s coefficient. The data are presented as medians (interquartile range). *p < 0.05 in relation to the control (CTL) group.

We investigated whether rapamycin-induced proteinuria was associated with impaired protein reabsorption in the PTECs. The uptake of albumin-FITC in the renal cortex was analyzed. Albumin uptake was reduced in all treated groups in relation to controls (Figure 4A). In agreement with proteinuria and albuminuria, there was a marked decrease in albumin uptake in the D1 and D3 groups; albumin uptake was also decreased in the D7 group, although to a lesser degree.

Proximal tubule protein reabsorption is a process that mainly relies on receptor-mediated endocytosis (Christensen et al., 2012; Eshbach and Weisz, 2017). Therefore, we assessed the expression of megalin in PTECs by immunofluorescence using confocal microscopy to analyze if rapamycin modulates components of the receptor-mediated endocytosis pathway. A reduction of megalin expression was observed in all rapamycin-treated groups compared with controls (Figures 4B–E). There was an acute reduction of megalin expression in the D1 and D3 groups and a mild reduction of megalin expression in the D7 group.

**FIGURE 5:**
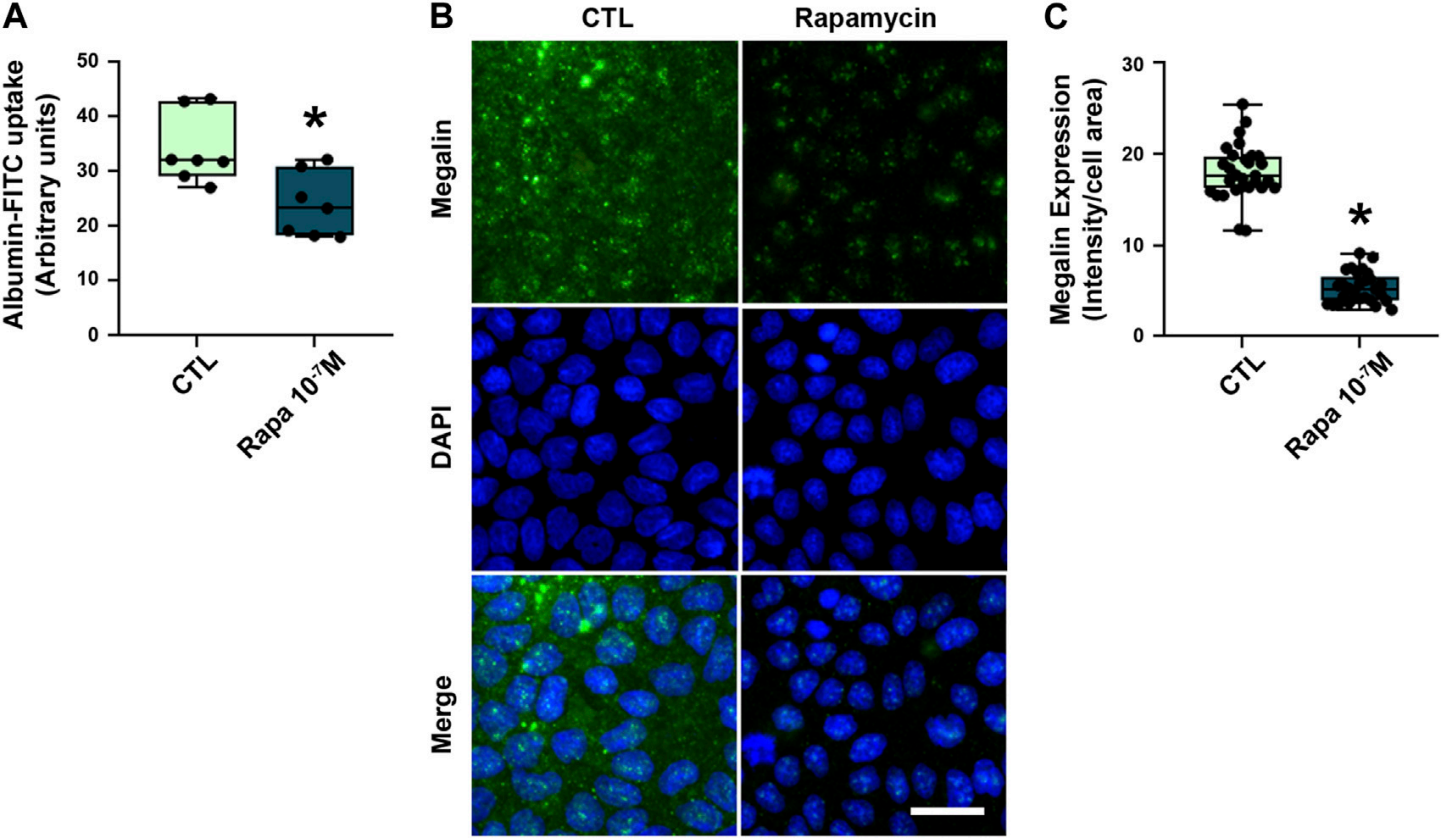
Rapamycin treatment decreased megalin-mediated albumin uptake in LLC-PK1 cells. **(A)** Albumin-FITC uptake in LLC-PK1 cells treated with 10^–7^ M rapamycin overnight (*n* = 7). **(B)** Representative confocal microscopy images of megalin expression (green). Cells nuclei were stained with 4′,6-diamidino-2-phenylindole (DAPI, blue). Scale bar, 50 µm. **(C)** Quantification of total megalin expression in LLC-PK1 cells (symbols represent randomly selected cells per field of three independent experiments, *n* = 3). Data are presented as medians (interquartile range). *p < 0.05 in relation to the control (CTL) group.

Besides the total expression of megalin in PT cells, its localization at the apical brush border membrane is essential for protein reabsorption (Marzolo and Farfán, 2011; Perez Bay et al., 2016). The expression pattern of megalin was mostly restricted to the brush border of PTECs in the control group (Figures 4C,D,F). Rapamycin treatment resulted in a decrease of megalin expression at this site. Furthermore, the distribution of megalin between the brush border and intracellular compartments was changed by rapamycin treatment (Figure 4G). The level of megalin in intracellular compartments in relation to the brush border increased from day 1 to day 7 after the treatment. These data indicate that rapamycin treatment changed both total megalin expression and cellular distribution. Furthermore, a direct correlation between albumin uptake and megalin expression was found in all rapamycin-treated groups, and there was an inverse correlation between albumin uptake and UPCr or 24-h proteinuria (Figures 4H–J). To confirm the direct effect of rapamycin on the PTECs, we analyzed this parameter *in vitro* with LLC-PK1 cells. Overnight rapamycin treatment (0.1 mM) decreased albumin-FITC uptake and megalin expression (Figures 5A–C).

## 4 Discussion

The therapeutic use of rapamycin after solid organ transplantation and for autoimmune diseases has become an alternative to other conventional immunosuppressive therapies (Johnson et al., 2001; Weir et al., 2011; Tedesco-Silva et al., 2019; Peng et al., 2020). However, the development of side effects including proteinuria and albuminuria in patients and murine models is an important limitation of rapamycin use (van den Akker et al., 2006; Aliabadi et al., 2008; Wiseman et al., 2013; Tedesco-Silva et al., 2019; Asleh et al., 2021). In the present study, a possible mechanism underlying the proteinuria and albuminuria induced by rapamycin was revealed. We showed that rapamycin reduces megalin-mediated protein endocytosis in PTECs in healthy BALB/c mice. This effect was associated with a decrease in megalin expression in PTECs without changes in glomerular function and structure, indicating a tubular origin of the proteinuria and albuminuria.

One important concern could be the use of creatinine clearance (CCr) as a marker of glomerular function since creatinine is secreted in PTECs (Eisner et al., 2010; Vallon et al., 2012). The gold standard for measuring absolute glomerular flow rate (GFR) is the clearance of inulin. Vallon et al. (2012), using C57Bl/6 mice, demonstrated that creatinine clearance was higher than inulin clearance. However, when creatinine secretion in the proximal tubule was abolished in knockout mice for organic anion transporters (OAT1 and OAT3), the values became similar. On the other hand, Dunn et al. (2004), using C57Bl/6 mice, showed that GFR measured by inulin clearance was very similar to that obtained by CCr using HPLC method. Despite these contradictory results, CCr has been widely used by several authors as a marker of glomerular function in experimental animal model procedures and human clinical tests (Liu et al., 2012; Barnett et al., 2022; Corremans et al., 2022). Due to the endogenous nature of creatinine, the use of CCr to assess glomerular function avoids invasive procedures. In our study, we did not observe any changes in the urinary excretion of creatinine and estimated GFR (eGFR), indicating that there were no modifications in the secretion of creatinine. Therefore, these observations support the use of CCr as a reliable estimate of glomerular function in our experimental condition. Furthermore, the CCr values obtained in the present study were similar to those reported in other works (Abreu et al., 2014).

Stylianou et al. (2012) observed that rapamycin treatment of healthy female BALB/c mice with 1.5 mg/kg/day rapamycin for 1 week, presented a slight increase in albuminuria. A more pronounced effect was observed only when the animals received 3.0 mg/kg/day of rapamycin. In addition, the authors showed transient peak in albuminuria after 4 weeks of rapamycin treatment (1.5 mg/kg/day) followed by regression at 8 weeks. We showed that treatment of healthy male BALB/c mice with the same concentration and time of treatment was associated with transient proteinuria and albuminuria that was higher after 3 days of treatment than after 7 days. These results point to a dynamic and transient effect of rapamycin on the development of proteinuria, depending on treatment duration and dosage. Furthermore, our results revealed that rapamycin treatment induces a specific effect on the renal handling of protein since it did not apparently change renal sodium handling as well as cortical (Na^+^+K^+^)ATPase activity. In agreement, rapamycin treatment did not change phosphorus/bicarbonate excretion in asymptomatic kidney transplant patients (Banhara et al., 2015).

Despite this evidence, the effect of rapamycin treatment on renal sodium handling is a controversial matter. Haller et al. (2012) showed that rapamycin treatment (1.5 mg/kg/day) of healthy Wistar rats for 7 days was associated with increased urinary excretion of Na^+^. However, similar to our results, they did not observe any modification in the expression of the (Na^+^+K^+^)ATPase α1subunit in the renal cortex. Therefore, the effects of rapamycin on renal sodium handling may not be associated with modulation of the activity and expression of (Na^+^+K^+^)ATPase in PTECs. On the other hand, the urinary excretion of Na^+^ was reduced after rapamycin treatment (0.4 mg/kg/day, subcutaneously) in Sprague-Dawley rats (Nielsen et al., 2005). These contradictory results indicate that the effect of rapamycin depends on the dose, time of treatment, and route of administration as well as gender, strain, and species (Di Joseph and Sehgal, 1993; Nielsen et al., 2005; Haller et al., 2012; Stylianou et al., 2012). Furthermore, the specific modulation of sodium cotransporters in proximal and distal nephron segments is not ruled out. In fact, studies have shown that megalin expression modulates the expression of the Slc34a1 gene, which encodes the NaPi cotransporter (Weisz, 2021; Rbaibi et al., 2023). Mutations in megalin have been associated with Donnai-Barrow syndrome, a condition characterized by severe impairment of proximal tubule reabsorption (Kantarci et al., 2007). However, further investigations are necessary to fully elucidate this issue.

In a case report of a renal transplant recipient, Straathof-Galema et al. (2006) described nephrotic proteinuria due to use of rapamycin. Using renal biopsy and the FITC-labeled anti-albumin technique, the authors demonstrated a reduction in albumin reabsorption in PTECs. Proteinuria returned to normal levels after the withdrawal of rapamycin, indicating a transient effect. Here, we showed that albumin uptake in PTECs is decreased by rapamycin treatment in both an animal model and a cell culture. Furthermore, the inhibitory effect of rapamycin on protein uptake in immortalized PT cell lines has been reported (Oroszlan et al., 2010; Grahammer et al., 2016; Peruchetti et al., 2018). These results show that rapamycin treatment may induce tubular proteinuria and albuminuria by decreasing protein uptake in PTECs without changes in glomerular function.

By using a genetic approach to inhibit mTORC1 in the PT, which is the expected pharmacologic effect of rapamycin on PTECs, Grahammer et al. (2016) observed that transgenic mice presented a Fanconi-like syndrome and albuminuria due to a reduction in fluid-phase and receptor-mediated endocytosis. The authors suggest that this finding was associated with impaired intracellular trafficking and accumulation of endocytosed albumin despite preserved expression of megalin and cubilin in PTECs. In a previous work, our group showed that acute inhibition of the PI3K/PKB pathway, which promotes activation of mTORC1, halted albumin endocytosis in early endosomes (Silva-Aguiar et al., 2022a). The present work shows that rapamycin treatment for 1, 3, and 7 days reduced brush border expression and cellular distribution of megalin. In agreement, Gleixner et al. (2014) showed that prolonged rapamycin treatment (10 mg/kg/day for 6 months) induced proteinuria associated with decreased renal expression of megalin in C57BL/6 mice. This effect was mediated by transcriptional downregulation of megalin mRNA (Gleixner et al., 2014). In line with these observations, we propose that rapamycin may compromise megalin expression and cellular localization, which are essential for the proper functioning of receptor-mediated endocytosis pathway in PTECs (Marzolo and Farfán, 2011; Perez Bay et al., 2016; Silva-Aguiar et al., 2022a).

Changes in the protein reabsorption machinery in PTECs are usually associated with the development of tubule interstitial injury in subclinical AKI (Peruchetti et al., 2020; Peruchetti et al., 2021; Peres et al., 2023). However, we did not verify tubular injury in rapamycin-treated animals. In agreement, Stylianou et al. (2012) reported no evidence of tubular injury in female BALB/c mice treated with increasing doses of rapamycin (1.0, 1.5, and 3.0 mg/kg/day) for 7 days. Even higher doses of rapamycin (75 mg/kg/day) did not change tubule interstitial morphology in mice (Di Joseph and Sehgal, 1993). On the other hand, chronic rapamycin treatment induced cytoplasm vacuolization in the PT of C57BL/6 mice (Gleixner et al., 2014). Although it is not a specific histopathologic marker of kidney injury (Cristofori et al., 2007; Dickenmann et al., 2008; Ding et al., 2020), this finding could be a consequence of changes in intravesicular trafficking rather than changes in the ultrastructure of PTs. These data indicate that rapamycin treatment *per se* does not induce kidney injury despite modulation of protein reabsorption in PTECs.

Several studies have shown that the treatment of animal models affected by kidney disease with rapamycin ameliorates renal damage and proteinuria (Wu et al., 2006; Chen et al., 2012; Jiang et al., 2012; Xiao et al., 2014; Cui et al., 2015). Jiang et al. (2012) reported that rapamycin treatment (1.0 mg/kg) reduced tubular damage resulting from cisplatin-induced acute kidney injury in C57BL/6 mice. A similar effect was observed in a mouse model of tubule interstitial fibrosis and diabetic kidney disease (DKD) (Wu et al., 2006; Chen et al., 2012; Xiao et al., 2014). Xiao et al. (2014) showed that rapamycin treatment (2.0 mg/kg/48 h) reduced albuminuria observed in DKD, which is in contrast to the effect of rapamycin treatment in a healthy animal. It is well known that during the development of DKD, there is overactivation of mTORC1 in glomerular and tubular structures involved with kidney damage (Gödel et al., 2011; Inoki et al., 2011; Kogot-Levin et al., 2020). In addition, in a previous work, it was shown that the balance between the activities of mTORC1 and mTORC2 is a critical step for modulation of albumin endocytosis in PTECs (Peruchetti et al., 2014). Based on this evidence, we postulate that the possible success with the treatment of renal disease with rapamycin depends on the previous state of mTORC1 activation. Whether the beneficial effects of rapamycin on the tubular damage observed in DKD is associated with modulation of the megalin-mediated protein reabsorption in PTECs remains to be elucidated.

Our work has revealed an important action mechanism underlying the proteinuria induced by rapamycin treatment. This work demonstrates that rapamycin treatment induces proteinuria and albuminuria in healthy BALB/c mice without changes in parameters of glomerular function. This effect seems to be mediated by a specific decrease in megalin-mediated protein reabsorption in PTECs, involving a decrease in the expression and cellular distribution of megalin. These results open new perspectives on understanding the effects of rapamycin on the genesis of proteinuria.

## Data Availability

The original contributions presented in the study are included in the article/supplementary material, further inquiries can be directed to the corresponding author.

## Abbreviations

BSA, bovine serum albumin; BUN, blood urea nitrogen; DAPI, 4′,6-diamidino-2-phenylindole; DKD, diabetic kidney disease; DMEM, Dulbecco’s modified Eagle’s medium; FBS, fetal bovine serum; FITC, fluorescein isothiocyanate; LDH, lactate dehydrogenase; mTOR, mammalian target of rapamycin; mTORC1/2, mammalian target of rapamycin complex 1/2; PAS, periodic acid-Schiff; PI3K, phosphatidylinositol-3 kinase; PMSF, phenylmethylsulfonyl fluoride; PT, proximal tubule; PTEC, proximal tubule epithelial cells; UPCr, urinary protein to creatinine ratio.
